# First demonstration of left bundle branch area pacemaker implantation guided by artificial intelligence electrocardiographic mapping

**DOI:** 10.1016/j.hrcr.2025.03.001

**Published:** 2025-03-12

**Authors:** Omar Aldaas, Erin Dreessens, Jonathan C. Hsu, Ulrika Birgersdotter-Green, Gordon Ho, David E. Krummen

**Affiliations:** 1 University of California San Diego, La Jolla, California; 2 VA San Diego Healthcare System, San Diego, California

**Keywords:** Left bundle branch area pacemaker, Artificial intelligence, Noninvasive mapping, Electrocardiogram, Congestive heart failure

**Key Teaching Points**

• A case of a patient with severe contrast allergy and chronic kidney disease who presented for left bundle branch area pacemaker (LBBAP) implantation is presented.
• The first use of forward-solution electrocardiographic mapping to guide implantation of the LBBAP lead is demonstrated.
• LBBAP implantation without use of contrast and with reduced need for fluoroscopic imaging during LBBAP lead positioning on the right ventricular septum can be achieved.

## Introduction

The benefits of left bundle branch area pacemaker (LBBAP) implantation are becoming increasingly recognized^1^ in patients with high expected burdens of ventricular pacing, particularly in the presence of left ventricular dysfunction. Recent guidelines provide enhanced clarity regarding the populations for which LBBAP therapy may be most effective.^2^

When guiding the LBBAP lead to the appropriate site in the right ventricular (RV) septum, some laboratories use intravenous contrast injection to localize the septal sheath within the right ventricle to guide implantation.^3^ This use of intravenous contrast may place patients at increased risk of contrast-induced nephropathy,^4^ anaphylaxis, and increased procedural cost. Other providers perform implantation using fluoroscopic imaging and the paced QRS morphology to guide LBBAP lead positioning. This approach may expose operators to ionizing radiation during the LBBAP lead manipulation process and may provide less precise guidance, particularly in the presence of structural heart disease.

We report for the first time the use of artificial intelligence (AI)-enabled electrocardiographic (ECG) mapping^5^ to guide the implantation of a dual-chamber LBBAP device without contrast use and with decreased use of fluoroscopy during the LBBAP manipulation on the RV septum in an older adult patient with advanced chronic kidney disease and a severe iodinated contrast allergy.

## Case report

**Figure 1:**
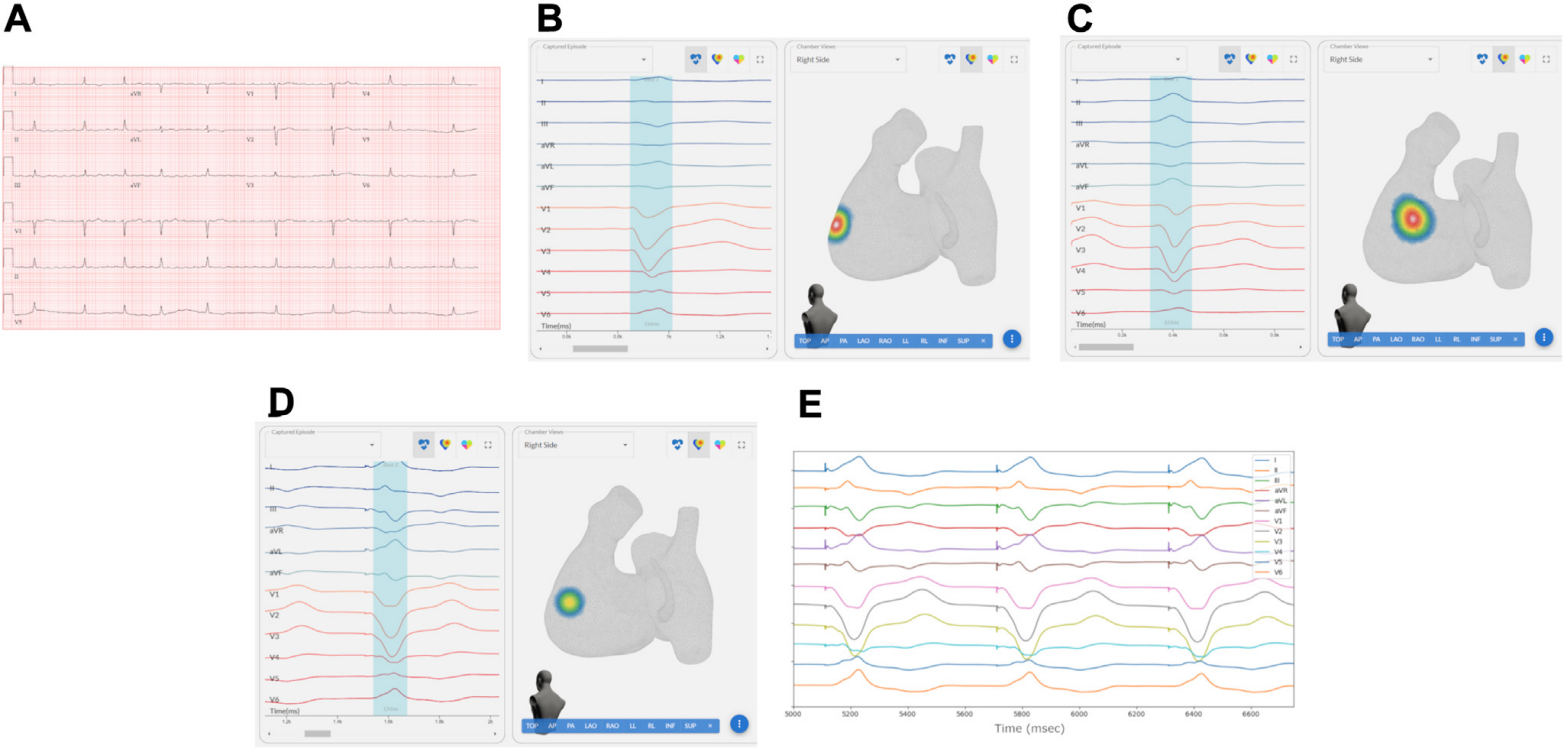
**A:** Electrocardiography (ECG) during arial fibrillation demonstrates a slow ventricular response of 53 beats/min associated with fatigue. **B:** Initial left bundle branch area pacemaker (LBBAP) pacing localization demonstrating apical positioning with respect to the target area. **C:** Second LBBAP pacing localization demonstrates that the lead is now superiorly displaced with respect to the target LBB area. **D:** After second repositioning, LBBAP lead pacing demonstrates activation of the target region of the right ventricular septum. **E.** ECG exhibiting characteristics of the target area, including lead II amplitude greater than III, aVR and aVL being discordant, and a notched V1.

A 90-year-old male patient with paroxysmal atrial fibrillation (AF), diabetes mellitus type 2, hypertension, hyperlipidemia, myocardial infarction status post percutaneous coronary intervention, heart failure with reduced ejection fraction, obstructive sleep apnea, chronic kidney disease stage IIIb, and a severe iodinated contrast allergy presented with fatigue, heart failure exacerbation, and AF with symptomatic bradycardia at a rate of 53 beats/min (Figure 1A). Laboratory evaluation revealed a serum blood urea nitrogen of 29 mg/dL and a creatinine of 2.01 mg/dL with an estimated glomerular filtration rate of 31 mL/min. Echocardiography revealed a left ventricular ejection fraction of 30.6%. Prior nuclear sestamibi imaging demonstrated a stable inferior transmural infarction with no reversible perfusion defects. After diuresis and medical optimization, the patient underwent electrical cardioversion. Subsequent telemetry monitoring demonstrated sinus bradycardia and the patient remained fatigued and lightheaded. We therefore initiated a discussion regarding device therapy with the patient and his family. Given the patient’s history of recurrent AF events and chronic need for beta adrenergic blockers as guideline-directed medical therapy for his heart failure with reduced ejection fraction, we were concerned that his need for ventricular pacing^2^ would be >20%–40%, as AF often becomes more frequent over time. The patient refused an implantable cardioverter-defibrillator, given his advanced age and personal preference. After a shared medical decision-making process, the patient and medical team decided that a dual-chamber LBBAP would provide protection from bradycardia during future episodes of AF and reduce the risk of pacing-induced cardiomyopathy, given his already-reduced left ventricular ejection fraction.

To reduce the risk of contrast-induced nephropathy^4^ and anaphylaxis and reduce fluoroscopy use during LBBAP lead positioning, a novel workflow was developed to use a noninvasive AI-based^6^ mapping system (vMap, Vektor Medical, La Jolla, CA) to iteratively guide mapping of the LBBAP tip on the RV septum.

### AI Mapping and Procedural Details

Before the implantation procedure, information regarding the patient’s anatomy, including the presence of the inferior/posterior left ventricular scar, was entered into the AI mapping system using drop-down menus. Next, the patient was sedated under monitored anesthesia care and access to the left axillary vein was accomplished using ultrasound guidance. A hydrophilic wire was advanced to the pulmonary artery and a septal sheath was advanced over the wire into the RV outflow tract.

The septal sheath was retracted into the right ventricle and the LBBAP lead (3830 SelectSecure, Medtronic, Minneapolis, MN) was advanced to the tip of the sheath to allow pacing. Pacing wires were connected to the lead tip and to an instrument within the pocket for unipolar stimulation. Pacing was initiated at an output of 5 V at 2-ms duration.

The paced QRS morphology observed on the electrophysiologic recording system (Bard, Boston Scientific, Marlborough, MA) at the initial lead location was saved to a USB memory device. The memory device was then transferred to the forward solution AI mapping computer. The AI system can identify and highlight QRS complexes automatically; the paced QRS of interest was selected for analysis by clicking on the blue region containing the target QRS complex (Figure 1B, left side). The pacing site was then visualized on the 3-dimensional cardiac model (Figure 1B, right side).

The initial mapping demonstrated that the LBBAP was apically displaced relative to the target area and the lead was pulled back. The paced QRS morphology at the second location was selected (Figure 1C, left) and mapped (Figure 1C, right). The lead tip was now too high in the right ventricle; the sheath and lead were again repositioned on the RV septum.

At the next location, the paced QRS complex met criteria for positioning within the target zone^3^ (Figure 1E), with lead II greater than III, aVR and aVL discordant, and a notched V1. The lead was then rotated to penetrate the ventricular septum.

**Figure 2:**
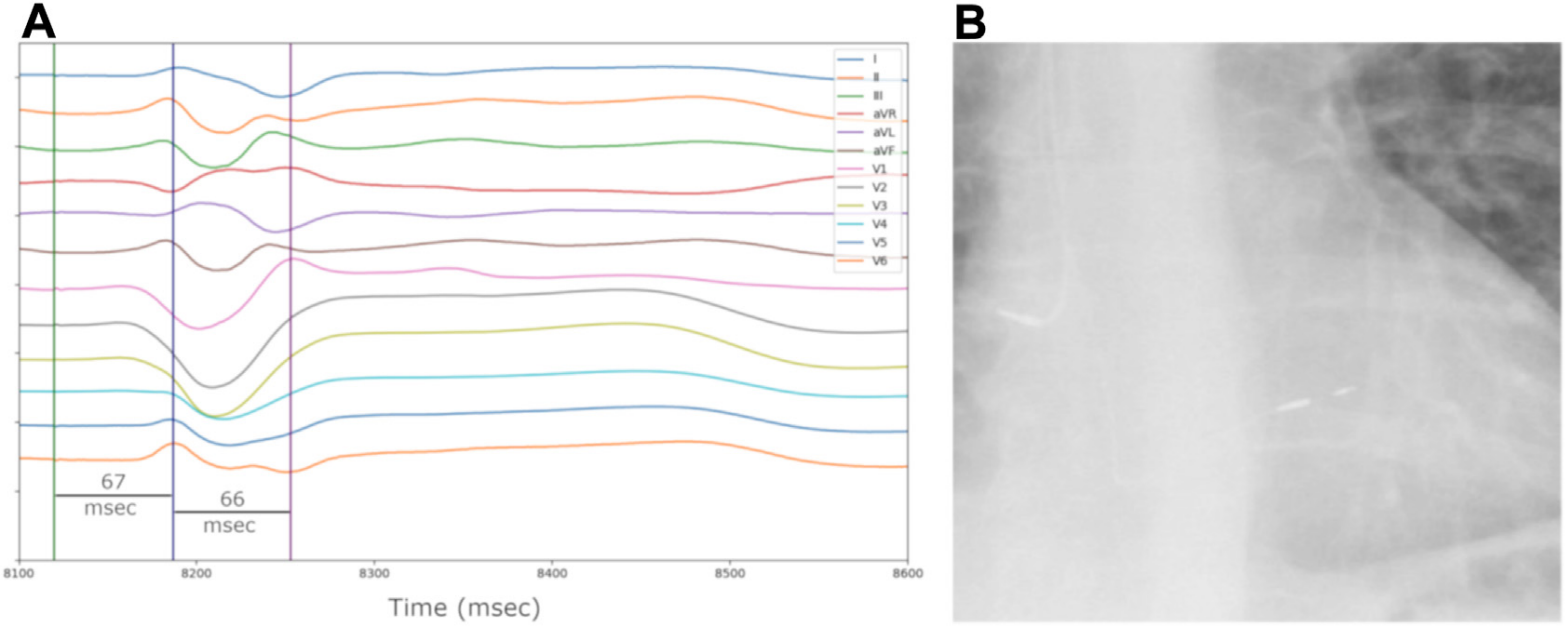
**A:** Electrocardiography (ECG) at final lead position showing QRS morphology at low pacing output. The stimulus (*green vertical line*) to peak R in V6 (*dark blue vertical line*) is 67 ms, and the peak R in V6 to peak R in V1 (*purple vertical line*) is 66 ms, consistent with left bundle branch area pacing (LBBAP). **B:** Radiograph showing final LBBAP lead and right atrial lead positions.

The lead was incrementally rotated and advanced until the impedance decreased, and a positive terminal deflection was seen on lead V1. At the final lead location, time from stimulus to R peak in lead V6 was 67 ms, meeting the criteria for LBB capture (Figure 2A).

The lead was then tested showing acceptable sensing, impedance, and thresholds. The septal sheath was removed with a cutting tool and the lead anchored to the pectoralis major muscle with nonabsorbable suture. The right atrial lead and pulse generator were implanted per standard clinical protocol. The case was successfully concluded without the use of contrast and with reduced need for fluoroscopy during LBBAP manipulation. Fluoroscopy was still used to assess sheath angulation during advancement of the lead, assess lead slack during and after sheath splitting, and guide right atrial lead implantation; total fluoroscopy use was 7.6 minutes. Final lead positions are shown in Figure 2B.

Interrogation at 2 weeks’ follow-up demonstrated acceptable LBB lead sensing (4.4 mV), impedance (608 Ω), and thresholds (0.625 V at 0.4 ms) and the patient felt well, with a reduction in heart failure symptoms.

## Discussion

Cardiac physiologic pacing has been found to reduce progression to heart failure and death.^7^ The LBBAP approach^2^^,^^8^ has demonstrated multiple advantages compared with His bundle pacing, including improved ventricular sensing and lower pacing thresholds.^9^ Compared with RV apical pacing, LBBAP pacing carries a lower risk of pacing-induced cardiomyopathy and risk of AF,^1^ particularly in patients with high expected burdens of ventricular pacing and reduced left ventricular ejection fraction.^2^

Disadvantages of the LBBAP vs RV apical pacing include increased technical complexity of the procedure and, for some operators, increased fluoroscopy use and injection of intravenous contrast to help visualize the location of the septal sheath within the right ventricle.^3^ Although generally safe and well-tolerated, use of contrast is associated with an increased risk of contrast-induced nephropathy,^4^ anaphylaxis, and increased costs. Although overall fluoroscopy exposure for operators in individual cases is relatively low, increasing awareness of the risks of recurrent fluoroscopy exposure has generated significant interest in additional technologies and techniques to further reduce exposure to ionizing radiation.^10^

In this report, we described for the first time the use of a noninvasive ECG-based mapping system to guide positioning of the LBBAP lead on the RV septum. The AI mapping system compares the patient’s paced QRS morphology with a digital reference library consisting of thousands of cardiac simulations of RV pacing and high-quality clinical data to localize the lead tip within the heart^5^; it is currently US Food and Drug Administration–cleared for analysis of atrial and ventricular pacing. Using this technology in an iterative fashion, the lead was able to successfully be positioned within the target area with subsequent evidence of LB capture.

In the absence of the AI mapping technology, implantation of a LBBAP lead would be accomplished using both fluoroscopy and interpretation of the paced QRS morphology (manual pace mapping). There are several advantages to using the AI mapping system for LBBAP lead implantation over manual pace mapping. One advantage is an improved understanding of the spatial location of the tip of the LBBAP lead within the right ventricle. This may provide more concrete feedback regarding the manipulations required to maneuver the LBBAP lead tip into the optimal position, particularly for less experienced operators. This may also reduce the need for fluoroscopic imaging of the LBBAP lead during the RV septal mapping process. This is important from a radiation safety perspective, as LBBAP lead implantation is often performed with the physician close to the fluoroscopic imaging field. Although fluoroscopy was still used to evaluate sheath angulation during the lead advancement process, assess lead slack after implantation,^11^ and guide right atrial lead implantation, future workflows may allow further reductions in radiography use, such as incorporation of transthoracic echocardiographic imaging.^12^

Another advantage of the technology is to store each of the positions of the LBBAP lead tip and the associated paced QRS morphology during RV septal mapping. This can be helpful in several situations, including cases in which the “ideal” pacing site is not encountered in the expected spatial location (as occurred in this case) of 1.0–1.5 cm apical from the bundle of His^3^; it allows the user to rapidly understand what sites have been assessed and efficiently expand the search to accelerate the mapping process. In the described case, the desired electrogram morphology occurred somewhat more apical than expected, although this position is consistent with recent work from Vijayaraman and colleagues,^12^ who used echocardiographic imaging to assist and assess LBBAP implantation. In that study, the mean ± SD distance from the tricuspid valve to the site of successful LBBAP implantation was 27 ± 7 mm.

The technology can also be helpful for patients in whom the “ideal” electrogram morphology is not encountered, even after an expanded search. In prior work, Padala and colleagues^13^ noted that a “notch” or “w” pattern in V1 was seen in only 57.6% of LBBAP cases (90 of 156 patients). Because paced 12-lead ECG morphology remains the arbiter of lead advancement in this workflow, the AI mapping system allows recall of all paced QRS morphologies and pacing locations to rapidly select a “best candidate site” for lead implantation and testing.

A current disadvantage of using the AI mapping system regards the need to transfer the digital ECG data from the electrophysiologic recording system to the AI mapping system using a USB memory device. Future versions of the AI mapping technology are planned to allow continuous streaming of the ECG data to the mapping system, enabling near real-time mapping results.

In prior blinded, multicenter, independently adjudicated work, the algorithm demonstrated a spatial accuracy of 9 mm (95% CI, 7–17 mm) for ventricular pacing.^5^ Future studies are required to test the hypothesis that this approach may lead to decreased fluoroscopy use and shorter procedure times vs controls. If validated, this workflow may help to reduce risks associated with ionizing radiation exposure, which is particularly important for pediatric and pregnant patients.

## Conclusion

In this report, we described the first use of forward-solution AI-based ECG mapping to guide LBBAP implantation, resulting in successful LBBAP lead positioning and implantation.

## Disclosures

Dr. Hsu reports receiving honoraria from Medtronic, Abbott, Boston Scientific, Biotronik, Janssen Pharmaceuticals, Bristol-Myers Squibb, Pfizer, Sanofi, Hillrom, Baylis Medical, Acutus Medical, iRhythm, Zoll Medical, and Biosense-Webster; research grants from Biotronik and Biosense-Webster; and has equity interest in Vektor Medical. Dr Green reports equity in Vektor Medical. Dr Ho and Dr Krummen report equity in Vektor Medical and intellectual property, assigned to UCSD. Dr Krummen also reports consulting for Vektor Medical under an Institutional Consulting Agreement administered by UCSD. Dr Aldaas and Dr Dreessens report no conflicts of interest.
